# Discovery of porcine maternal factors related to nuclear reprogramming and early embryo development by proteomic analysis

**DOI:** 10.1186/s12953-015-0074-5

**Published:** 2015-06-27

**Authors:** Qi Zhao, Zheng Guo, Shanhua Piao, Chunsheng Wang, Tiezhu An

**Affiliations:** College of Life Science, Northeast Forestry University, 26 Hexing Road, Xiangfang Dist., Harbin, Helongjiang 150040 China

**Keywords:** Proteomics, IVF, Nuclear transfer, Maternal factors

## Abstract

**Background:**

Differentiated cell nuclei can be reprogrammed to a pluripotent state in several ways, including incubation with oocyte extracts, transfer into enucleated oocytes, and induced pluripotent stem cell technology. Nuclear transfer-mediated reprogramming has been proven to be the most efficient method. Maternal factors stored in oocytes have critical roles on nuclear reprogramming and early embryo development, but remain elusive.

**Results:**

In this study, we showed most of porcine oocytes became nuclear matured at 33 h of IVM and the rate had no significant difference with oocytes at 42 h of IVM (*p* > 0.05). Moreover, the cleavage and blastocyst rates of SCNT and PA embryos derived from 42O were significantly higher than that of 33O (*p* < 0.05). But 33O could sustain IVF embryo development with higher cleavage and blastocyst rates comparing to 42O (*p* < 0.05). To clarify the development potential difference between 33O and 42O, 18 differentially expressed proteins were identified by proteomic analysis, and randomly selected proteins were confirmed by Western blot. Bioinformatic analysis of these proteins revealed that 33O highly synthesized proteins related to fertilization, and 42O was rich in nuclear reprogramming factors.

**Conclusions:**

These results present a unique insight into maternal factors related to nuclear reprogramming and early embryo development.

**Electronic supplementary material:**

The online version of this article (doi:10.1186/s12953-015-0074-5) contains supplementary material, which is available to authorized users.

## Introduction

Reprogramming of sperm or somatic cells into pluripotency has great attraction and value in both research and therapy. Recent advances in iPS have clearly indicated that differentiated cells can acquire pluripotency by overexpressing a small number of transcript factors, such as *Oct4*, *Sox2*, *Klf4*, and *c*-*myc* [1]. However, the reprogramming process is slow (at least 1 to 2wk), and the efficiency is low (typically about 1 %) [2]. In contrast, reprogramming by oocytes occurs within one or two cell cycles, and often in a majority of embryos, suggesting oocyte-mediated reprogramming resets differentiated cell states more efficiently than the iPS method and more factors must participate in the process [3–5]. So, understanding of these oocyte factors will provide us important information on nuclear reprogramming and embryo development.

Given how little is known about the factors in mammalian oocyte, there are several recent reports utilizing proteomics approaches to explore the oocyte proteomes in cattle, pig and mouse [6–15]. For example, Calvert et al. identified 8 highly abundant HSPs and related chaperones in the mature mouse egg by two-dimensional electrophoresis [16]. Vitale et al. used 2DE and MS to identify 12 proteins that appeared to be differentially expressed between GV and MII murine oocytes [12]. However, it has some limitations in identifying proteins that are related to nuclear reprogramming and early embryo development. So, a comparison of the proteomes of oocytes with different abilities of nuclear reprogramming and development may aid in the identification of the maternal factors.

In the study, we checked the effect of oocyte maturation time on early development of IVF, PA and SCNT embryos. Moreover, mass spectrometry was applied to identify the maternal factors related to nuclear reprogramming and early embryo development, and we successfully discovered 18 differentially expressed proteins between 33O and 42O in pig.

## Materials and methods

### IVM of porcine oocytes

Porcine ovaries were collected from a local slaughter house and kept in saline at 32–37 °C. Antral follicles (3–5 mm indiameter) were aspirated with an 18-gauge needle. Aspirated oocytes with an evenly granulated cytoplasm and at least three uniform layers of compact cumulus cells were selected and cultured in four-well plates (Nunc, Naperville, IL, USA) containing 500 μL of maturation medium that was TCM199 (Gibco) based medium plus 0.05 μg/ml EGF, 0.5 μg/ml LH and FSH, at 39 °C in 5 % CO_2_ in air [17]. The rates of polarbody extrusion were calculated from 16 h–42 h of IVM. Matured porcine oocyte was obtained at 33 h and 42 h for further experiments.

### IVF and cloned embryos production and in vitro culture

For IVF, freshly ejaculated sperm-rich fractions were collected from fertile boars, and following a short incubation at 38.5 °C, the semen was resuspended and washed three times in Dulbecco’s Phosphate Buffered Saline (DPBS) supplemented with 0.1 % (w/v) bovine serum albumin (BSA) by centrifugation at 1500 × g for 4 min. The spermatozoa concentration was measured using a hemocytometer, and the proportion of motile sperm was determined. The spermatozoa were diluted with modified Tris-buffered medium (mTBM) to an optimal concentration. Cumulus-free oocytes at 33 h or 42 h of IVM were washed three times in mTBM. Approximately 30 oocytes were inseminated in 50-ml drops of mTBM at a final sperm concentration of 3 × 10^5^ /ml for 6 h.

After 12 h co-incubation with spermatozoa, oocytes were fixed for 30 min in 4 % paraformaldehyde, and permeabilized for 20 min in 0.1 % triton X 100 following wash in PBS. Then oocytes were stained with 10 μg/ml Hoechst 33342 to visualize the nuclei. Samples were observed and photographed by using a Nikon Eclipse 80i epifluorescence microscope (Nikon, Tokyo, Japan). Oocytes with one or more swollen sperm head(s) and/or male pronuclei were considered as fertilization and fertilization rate was evaluated.

The procedure for porcine SCNT has been described previously [18]. After 33 h or 42 h of IVM, the oocytes were treated with 1 mg/ml hyaluronidase (H3506, Sigma-Aldrich, Missouri, USA) to remove the surrounding cumulus cells. Oocytes with a clearly extruded first polar body were selected as recipient cytoplasts. Cumulus-free oocytes were enucleated by aspirating the first polar body and adjacent cytoplasm with a glass pipette 25 mm in diameter in TCM199-Hepes plus 0.3 % BSA and 7.5 mg/ml Cytochalasin B. Porcine ear fetal fibroblasts at passage 5 served as donor cells and were injected into the perivitelline space of enucleated oocytes. Injected oocytes were placed in fusion/activation medium (0.3 M mannitol, 1.0 mM CaCl_2_, 0.1 mM MgCl_2_, and 0.5 mM HEPES). Fusion/activation was induced with 2 DC pulses of 1.2kv/cm for 30 msec on a BTX Elector-Cell Manipulator 2001 (BTX, San Diego, CA). Cumulus-free oocytes were directly activated by the same parameters as SCNT procedure for producing PA embryos.

The embryos were cultured in porcine zygote medium-3 (PZM-3) at 39 °C in 5 % CO_2_ in air. The cleavage and blastocyst rates were assessed at 48 and 156 h after activation, and the number of blastocyst cells was examined by nuclear staining with 5 μg/ml Hoechst 33342.

### Oocyte collection and proteomic analysis

Zona pellucida of more than 10,000 33O and 42O were removed (Additional file 1: Figure S1) and total proteins were extracted using ultrasonic wave and lysis buffer. The lysis buffer consisted of 7 M urea, 2 M thiourea, 4 % (w/v) CHAPS, 65 mM DTT, 2 % (v/v), and 1 % (v/v) protease inhibitor cocktail. The protein concentration was determined by the Bradford method and pH was adjusted to 8.5 with 50 mM NaOH. In fluorescent 2D DIGE, protein from 33O and 42O were equally pooled together and labeled with Cy2 as internal standard, and the two samples were labeled with Cy3 or Cy5, separately. Labelled samples were mixed in the rehydratation buffer before to be loaded onto the into 24-cm pH 3–10 IPG strips (Bio-Rad) and run in a single two-dimensional gel. Then, the gels were scanned using the Typhoon 9410 scaner with excitation/emission wavelengths of 488/520 nm for Cy2, 532/580 nm for Cy3 and 633/670 nm for Cy5. Image analysis were performed by Decyder software suite 5.02 (GE Healthcare) which allow the comparison of the different combination corresponding to the experimental conditions. An independent t-test was used to determine the significance between the experimental groups. P-values less than 0.05, and fold-changes greater than 1.5 were considered statistically significant.

For protein identification, the spots of interest were cut from the gels, and washed with 25 mM NH_4_HCO_3_ and 50 % ACN solution and dehydrated with 100 % ACN sequentially and dried by centrifugal lyophilization. The gels were digested with 15-20uL of 0.01ug/ul trypsin (Promega) in 25 mM ammonium bicarbonate for 15 h at 37 °C. The supernatants were collected, and the tryptic peptides were extracted from the gel sequentially with 5 % TFA at 40u for 1 h and with 2.5 % TFA, 50 % ACN at 30 °C for 1 h. The extracts were pooled and completely dried by centrifugal lyophilization. Digested peptides with matrix (50 % acetonitrile, 0.1 % TFA containing 3 mg/ml alpha-cyano-4-hydroxy cinnamic acid matrix) were then spotted on the target plate. Samples were analyzed by MALDI-TOF-TOF MS (4800 Proteomics Analyzer, Applied Biosystems) in positive reflectron modeat fixed laser fluency with low mass gate and delayed extraction. Database searching was carried out using Mascot version 2.2 (MatrixScience, London, UK) via GPS explorer software (ABI) version 3.6 combining MS and MS/MS interrogations on NCBI pig proteins database. In the searching parameter, modification was set as carbamido-methylation, oxidation, and a maximum of one missed trypsin cleavage was permitted. Tolerance of precursor and fragment ions were both set to 0.2 Da. The reported proteins were always those with the highest number of peptide matches. All identified proteins were those with a statistical significance (*p* ≤ 0.05) and the best ion score.

### Quantitative realtime PCR

Total RNA was extracted from at least 100 oocytes using the PureLink^TM^ Micro-to-Midi system (Invitrogen) according to the manufacturer’s instructions, and reverse transcription was used to generate cDNAs using the PrimeScript^TM^ RT Reagent Kit (TaKaRa). Real-time PCR was performed using SYBR Premix Ex Taq^TM^ (TaKaRa) and the 7500 Real-Time PCR System (Applied Biosystems). The reaction parameters were 95 °C for 30 sec, followed by 40 two-step cycles of 95 °C for 5 sec and 60 °C for 34 sec. Primers for each gene were listed (Additional file 1: Table S1). 18S rRNA was used as a reference gene. C_t_ values were calculated using Sequence Detection System software (Applied Biosystems), and the amount of target sequence normalized to the reference sequence was calculated as 2^–△△Ct^.

### Western blot

Oocytes removed zona pellucida, stored at −80 °C, were transferred to 10 μl cold 40 mM sodium phosphate, pH 7.6, containing 50 mM NaCl, 50 μM sodium orthovanadate, 10 mM sodium fluoride, 20 μM MG-132, 2 μM matrix metalloprotease inhibitor III (444264, Calbiochem, San Diego, USA), and 1 % protease inhibitor cocktail III (539134, Calbiochem, San Diego, USA). Homogenization was carried out at 4 °C with a Tekmar homogenizer by three 15 s bursts with a minute cooling in-between. Homogenates were centrifuged at 4 °C for 1 h at 100 000 × g. The supernatant solutions are referred to as “soluble” fractions. The pellets were suspended in the 0.2-0.25 ml complete buffer containing 1 % ASB-14 and were mixed every 15 min for 2 h with Radnoti glass pestles (Unitek, Monrovia, USA). After centrifugation at 4 °C for 1 h at 100 000 × g, the supernatants, referred to as “membrane extracts”, were removed and the pellets were discarded. About 50 oocytes of each soluble and membrane extract for each gene testing were separated by lithium dodecyl sulfate polyacrylamide gel electrophoresis on 4-12 % Bis-Tris NuPAGE gels and transferred to PVDF membranes (Invitrogen, Carlsbad, USA); nonspecific binding was blocked by overnight incubation in 1 % casein in PBS at room temperature. Blots were then probed for 2–4 h at room temperature with antibodys of PDIA3 (anti-PDIA3; ab13507, abcam, Cambridge, USA), SOD1 (anti-SOD1; ab13498, abcam, Cambridge, USA) and VIM (anti-VIM; V6630, Sigma, USA). Histone H2B (anti-H2B; ab40975, abcam, Cambridge, USA) served as loading control. After 2 h incubation at room temperature with secondary antibodies, protein bands were detected by enhanced chemiluminescence with the RPN2108 kit (Amersham, Piscataway, USA) and Kodak BioMax Light film.

### Statistical analysis

Statistical analysis was performed using SPSS 13.0 for MicroSoft^TM^ Windows. Data are shown as the mean ± SD. One-way ANOVA was used to assess any differences between groups. The Duncan method was employed for pairwise comparisons, followed by a Bonferroni correction. *p* < 0.05 (two-tailed) was considered statistically significant.

## Results

### Maturation of porcine oocytes during IVM

**Fig. 1:**
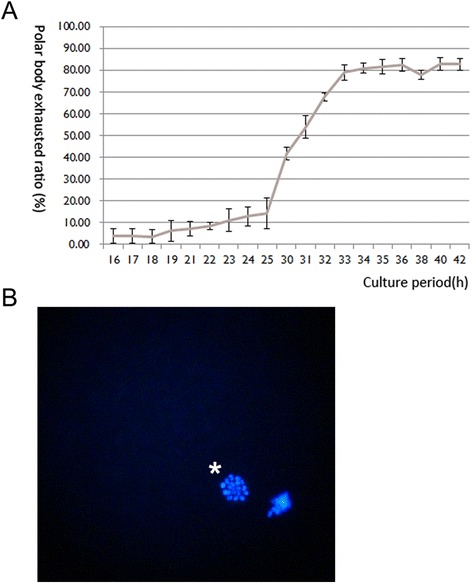
The porcine oocytes during IVM. **a** The polarbody extrusion during IVM of porcine oocytes. The polarbody extrusion mainly began at 25 h (14.45 %) of IVM, and the rate at 33 h reached the plateau. The rates of porcine oocyte polarbody extrusion at 33 h (76.72 %) and 42 h (81.80 %) of IVM had no significant difference (*p* > 0.05). **b** Fluorescence micrographs of nuclei in porcine MII oocytes (200×). DNA was blue, and the MII nuclei was marked by “*”

To get the time lapse characteristics of maturation of porcine oocytes during IVM, we detected the rate of 1^st^ polarbody extrusion from 16 h to 42 h. The oocyte began to extrude polarbody mainly at 25 h (14.45 %) of IVM and reached a plateau at 33 h (Fig. 1), and there was no significant difference between the rates at 33 h (76.72 %) and 42 h (81.80 %) of IVM (Additional file 1: Table S2; *p* > 0.05). We also found most of 33O (79.71 %) were arrested at the MII stage, and it had no remarkable difference with 42O (83.86 %; Table 1). Theoretically, the maturation quality of 42O must be better than 33O [17–21].

**Table 1:** The rates of oocytes 1^st^ polarbody extrusion at 33 h and 42 h of IVM

Culture period	No. of oocytes (repeats)	No. of MII oocytes (% ± SEM)
33 h	119 (3)	95 (79.71 ± 0.86)
42 h	125 (3)	105 (83.86 ± 0.76)

Highlights:

1. The majority of porcine oocytes achieve nuclear maturation at 33 h of IVM and proceed with cytoplasmic maturation from 33 h to 42 h

2. Porcine oocyte during 33 h of IVM is able to sustain IVF embryo development, and further cytoplasmic maturation is indispensable for development of SCNT and PA embryos

3. 18 differentially expressed proteins between oocytes during 33 h and 42 h of IVM are identified by mass spectrometry

### Effects of 33O and 42O on porcine embryo development

In order to test the effect of maturation time on nuclear reprogramming and embryo development, we employed 33O and 42O to produce IVF, SCNT and PA embryos (Table 2). The results showed the rates of cleavage and blastocyst of IVF embryos from 33O were significant higher than that from 42O (78.36 % vs 67.22 %, *p* < 0.05; 33.79 % vs 19.73 %, *p* < 0.05, respectively). Also, the rate of fertilization of 33O was significantly enhanced compared to 42O (85.19 % vs 78.09 %, *p* < 0.05; Additional file 1: Table S3). On the contrary, the rates of cleavage and blastocyst of SCNT embryos from 42O were significant higher than that from 33O (89.17 % vs 67.60 %, *p* < 0.05; 22.34 % vs13.54 %, *p* < 0.05, respectively), and the rates of enucleation of oocyte in both 33O and 42O reached up to 90 % (Additional file 1: Table S4), excluding the influence of enucleation on cloned embryo development. Moreover, the rates of cleavage and blastocyst of PA embryos from 42O were also significant higher than that from 33O (92.23 % vs 43.74 %, *p* < 0.05; 43.64 % vs 17.84 %, p < 0.05), and there was no significant difference of pronuclear rates at 6 h after artificial activation between 33O and 42O (Additional file 1: Table S5), suggesting lower development of 33O may be not simply due to lower activation compared to 42O. These results indicate that further maturation of oocyte after 1^st^ polarbody extrusion can accumulate maternal factors for SCNT and PA embryos development, but it may be not necessary for IVF embryo.

**Table 2:** The effect of 33O and 42O on IVF, NT and PA embryo development

Groups	Culture period	Repeats	No. oocytes	No. embryos fused	No. embryos cleaved	No. blastocysts	Total cell No. of blastocysts
				(% ± SEM)	(% ± SEM)	(% ± SEM)	(mean ± SEM)
IVF	33 h	5	314	−	247 (78.36 ± 1.19)a	106 (33.79 ± 1.89)a	39 ± 3 (n = 103)
	42 h	5	309	−	210 (67.22 ± 2.53)b	60 (19.73 ± 1.2)b	36 ± 5 (n = 56)
NT	33 h	4	411	280 (67.56 ± 4.71)	195 (67.60 ± 5.63)a	36 (13.54 ± 2.58)a	35 ± 2 (n = 33)
	42 h	4	750	467 (61.86 ± 4.68)	233 (89.17 ± 2.15)b	59 (22.34 ± 2.18)b	37 ± 2 (n = 57)
PA	33 h	7	537	−	232 (43.74 ± 2.62)a	88 (17.84 ± 2.06)a	36 ± 3 (n = 83)
	42 h	7	465	−	428 (92.23 ± 2.60)b	200 (43.64 ± 3.18)b	37 ± 4 (n = 96)

Values with different superscripts in the same group differ significantly (*P* < 0.05)

### Identification of maternal factors involved in nuclear reprogramming and embryo development

**Fig. 2:**
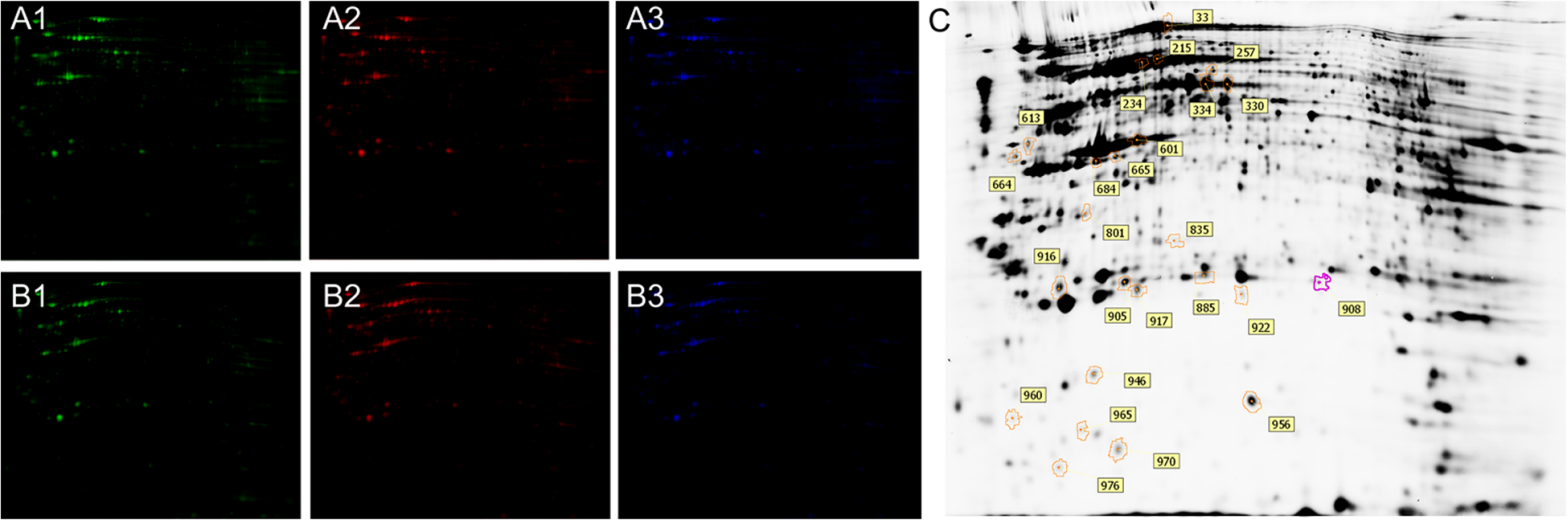
Total proteins seperated by 2D DIGE. Samples prepared from 33O (**a1, b2**) and 42O (**a2, b1**) were labeled with Cy3 or Cy5, separately, and the mixed samples were labeled with Cy2 for standard protein (**a3, b3**). The blue spots are internal standard proteins consisting of all samples labeled with Cy2. The differentially expressed protein spots between 33O and 42O were numbered in the 2D DIGE image (**c**)

**Fig. 3:**
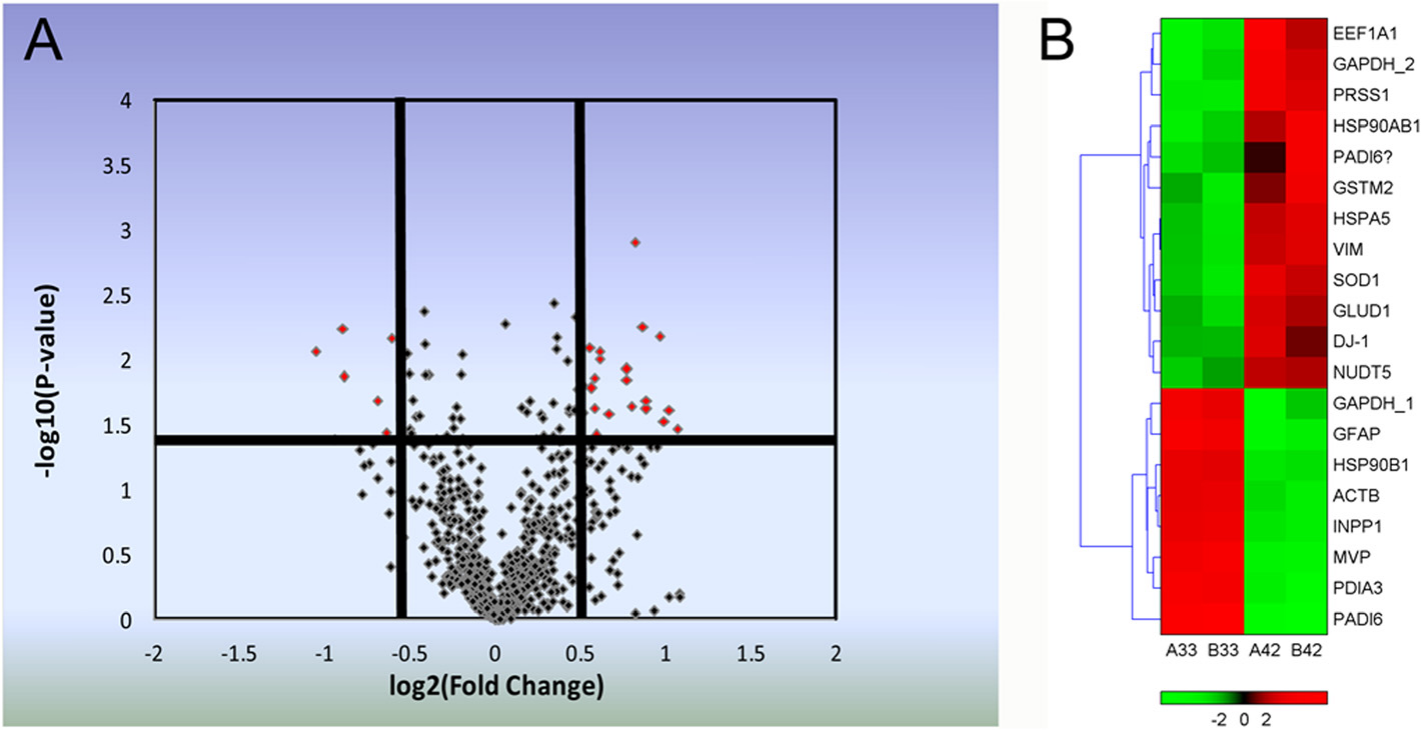
Differentially expressed proteins identified between 33O and 42O. **a** All protein spots (994) from 2D DIGE analysis. A horizontal axis represents the average log2 (ratio) of proteins (42O/33O), whereas a vertical axis represents the -log10 (P-value), P-value from t-test, red plots represent the protein spots which were significantly changed (fold > 1.5 and *p* < 0.05). **b** Heat map generated from 20 protein features selected in the 2D DIGE for analysis

**Fig. 4:**
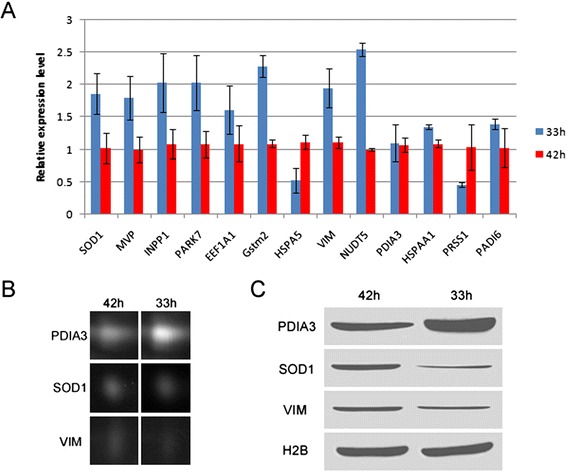
2D DIGE data validation. Though PCR results of 13 genes were inconsistent with the 2D DIGE results (**a**), the protein expression of 3 randomly selected genes (PDIA3, SOD1 and VIM) checked by Western blot (**c**) were in agreement with the gray values of these spots in the 2D DIGE (**b**). Histone H2B served as loading control

To uncover the basis for nuclear reprogramming and embryo development, global protein changes between 33O and 42O were examined by proteomic analysis. After oocytes collection and treatment, the total proteins were separated by 2D DIGE (Fig. 2). Analysis of the gel images showed 994 paired protein spots, and we used independent t-test to calculate differentially expressed proteins. We considered only spots with fold-changes greater than 1.5 to exclude the possibility of false positives by multiple comparisons. In total, 25 protein spots were found to be differentially expressed with fold-changes greater than 1.5 (*p* < 0.05; Fig. 3a), and finally 18 proteins were identified by MALDI-TOF/MS. Based on 2D DIGE analysis, 7 proteins were down-regulated and 11 proteins were up-regulated in comparison of 42O to 33O (Fig. 3b and Table 3). The 7 over-expressed proteins in 33O were indentified as protein-arginine deiminase type-6 (PADI6), major vault protein isoform 1(MVP), inositol polyphosphate-1-phosphatase (INPP1), glucose regulated protein 58 (PDIA3), glial fibrillary acidic protein (GFAP), 90-kDa heat shock protein (HSP90B1), and beta-actin(ACTG1). The 11 high expressed proteins in 42O were trypsinogen precursor (PRSS1), ADP-sugar pyrophosphatase-like isoform 1 (NUDT5), vimentin-like (VIM), heat shock 70 kDa protein 5 (HSPA5), heat shock 90kD protein 1 (HSP90AB1), eukaryotic translation elongation factor 1 alpha 1 (EEF1A1), similar to GLUD1 protein (GLUD1), glutathione S-transferasemu2 (GSTM2), superoxide dismutase [Cu-Zn] (SOD1), DJ-1 protein (PARK7), and glyceraldehyde-3-phosphate dehydrogenase (GAPDH). To comfirm the results, 2D DIGE data were examined by Quantitative realtime PCR and Western blot (Fig. 4). Though PCR results of 13 genes were inconsistent with the 2D DIGE results (Fig. 4a), the gray values of 3 randomly selected spots (PDIA3, SOD1 and VIM) in the 2D DIGE (Fig. 4b) were in agreement with the protein expressions of these genes checked by Western blot (Fig. 4c).

**Table 3:** Protein identifications by MS/MS analysis of oocytes which were significantly increased or decreased upon 42O, as compared with 33O

Spot no	Protein name	GI acession	Acession ID	Fold Diff.^a^	pl^b^	Mw^b^	Peptides matched	S cov %^c^	T-test (p)^d^	Classification
	Down-regulated									
1-2	PREDICTED: protein-arginine deiminase type-6	350585686	PADI6	−2.6/-1.73	5.45	79159.2	18	41.723	4.90E-02	enzyme
3	PREDICTED: major vault protein isoform 1	335284397	MVP	−1.85	5.57	99592.5	36	66.638	1.40E-02	Chaperone
4	inositol polyphosphate-1-phosphatase	350529419	INPP1	−1.61	5.21	44472.7	6	19.694	2.10E-02	enzyme
5-6	glucose regulated protein 58	301016769	PDIA3	−2.07/-1.91	5.93	56822.9	21	55.766	4.10E-02	oxidoreductase, isomerase
7	protein,glial fibrillary acidic	223869	GFAP	−1.86	5.96	24946.7	7	3.791	5.70E-03	Cytoskeleton
8	90-kDa heat shock protein	1945447	HSP90B1	−1.52	4.93	84721.7	27	42.224	6.80E-03	Chaperone
9	beta-actin	112980807	ACTG1	−1.55	5.5	29392.7	8	44.348	3.70E-02	Cytoskeleton
	Up-regulated									
10	glyceraldehyde-3-phosphate dehydrogenase	229279	GAPDH	1.96	6.9	35686.2	6	14.312	3.00E-02	Metabolism
11-12	DJ-1 protein	67038668	PARK7	1.94/1.52	6.33	19923.5	10	28.298	4.70E-02	Chaperone, oncogene, antioxidation
13	superoxide dismutase [Cu-Zn]	298677090	SOD1	1.74	6.03	15881.9	5	28.238	2.30E-02	Antioxidation
14	glutathione S-transferase mu 2	116047847	GSTM2	1.69	6.9	25746.9	4	4.67	4.80E-02	enzyme
15	PREDICTED: similar to GLUD1 protein	194042282	GLUD1	1.6	10.28	38321.5	13	8.87	2.60E-02	Metabolism
16	eukaryotic translation elongation factor 1 alpha 1	223019599	EEF1A1	2.08	9.1	50109.1	9	8.74	3.40E-02	enzyme/signal transducer
17	PREDICTED: heat shock 90kD protein 1, beta	194039391	HSP90AB1	1.86	4.96	83201.1	27	42.224	2.10E-02	Chaperone
18	PREDICTED: heat shock 70 kDa protein 5	194033595	HSPA5	1.71	5.68	73767.5	24	40.616	1.40E-02	Chaperone
19	PREDICTED: vimentin-like	76097691	VIM	1.71	4.79	34288.2	6	15.727	1.20E-02	Cytoskeleton
20	PREDICTED: ADP-sugar pyrophosphatase-like isoform 1	350589625	NUDT5	1.53	5.14	24253.2	4	31.635	1.00E-02	Metabolism
21	trypsinogen precursor	238866766	PRSS1	1.96	6.85	25864.6	3	10.866	6.60E-03	enzyme

^a^Fold change represents protein upregulate (+) or downregulate (−) in 42O compared with 33O

^b^Percentage sequence coverage (S cov %) if the mascot score was significant (*P* < 0.05)

^c^Theoretical isoelectric point (pI) and theoretical molecular weight (Mw)

^d^ *p*-value from t-test of the protein level between 33O and 42O

### Functional categorization and pathway analysis of the identified factors

**Fig. 5:**
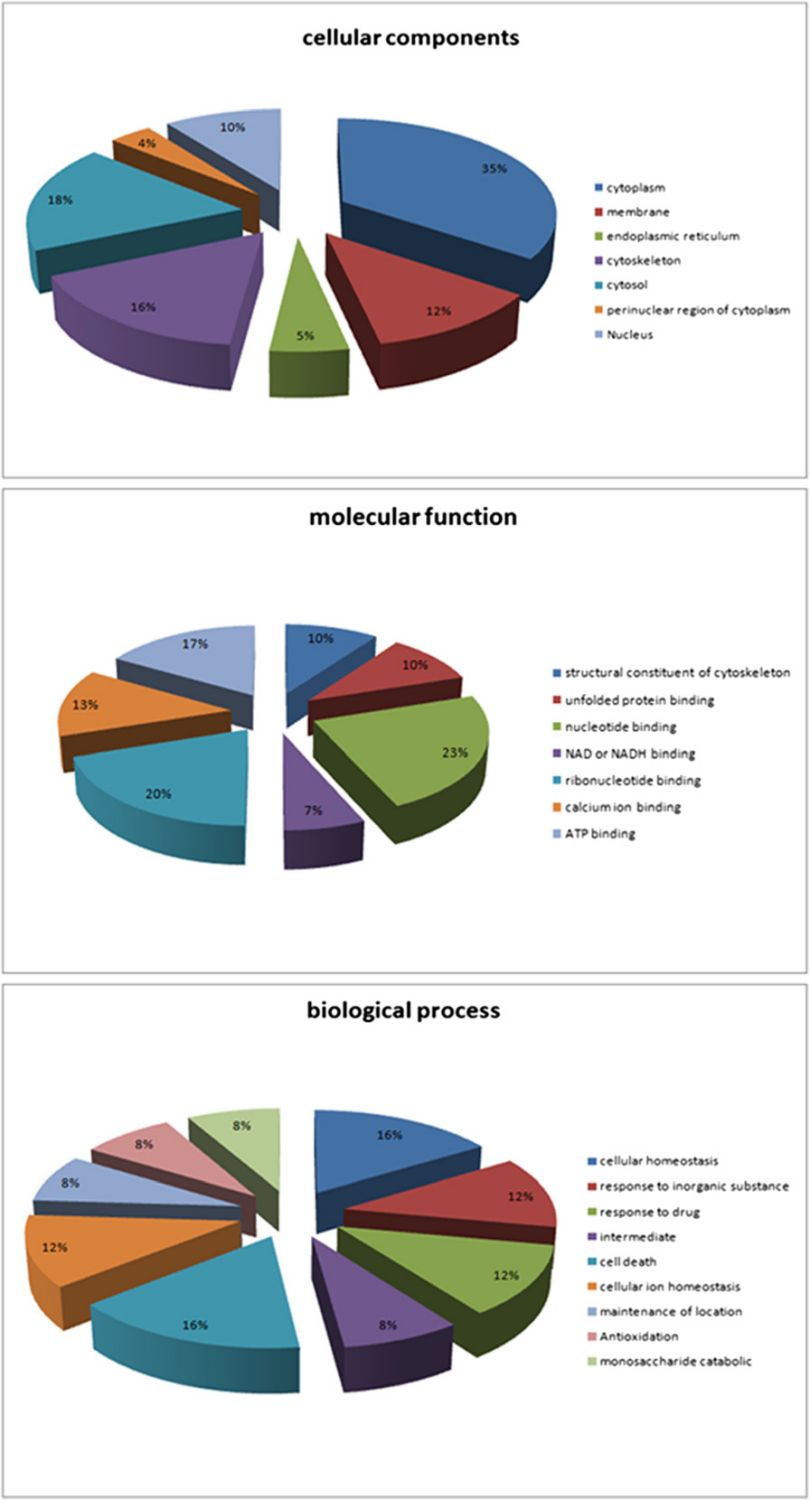
The classification of the identified proteins was performed according to the gene ontology term “molecular function”, “biological process” and “cellular component”

We categorized proteins identified in this study by searching Gene Ontology and performing a literature search. The differentially expressed proteins were classified into groups based on molecular function (structural constituent of cytoskeleton, unfolded protein binding, nucleotide binding, NAD or NADH binding, ribonucleotide binding, calcium ion binding and ATP binding), biological process (cellular homeostasis, response to inorganic substance, response to drug, intermediate, cell death, cellular ion homeostasis, maintenance of location, antioxidation and monosaccharide catabolic), and cellular component (cytoplasm, membrane, endoplasmic reticulum, cytoskeleton, cytosol, perinuclear region of cytoplasm and nucleus) with the UniProt database (Fig. 5). DAVID and Agilent Literature Search were combined by cytoscape to cluster most affected molecular functions in oocyte maturation, namely embryonic development, embryogenesis, ovulation, epigenetic modification, fertilization, meiosis, ageing, chromatin remodeling and anti-ageing. Additionally, we employed a detailed analysis of embryonic development, using automated text-mining tool which enables the software to generate pathways from entries in the PubMed database and Gene Ontology. These analysis revealed that proteins related to fertilization were found to be highly synthesized in 33O, such as PDIA3 [16], and nuclear reprogramming was significantly affected by high expressed proteins in 42O, such as PARK7 that was proved to be required for successful development of porcine SCNT embryos [22].

## Discussion

An increasing amount of data demonstrate that oocyte maturation involves major changes within the cytoplasmic compartment and these changes are essential for nuclear reprogramming and embryo development [14]. However, the molecular factors remain largely unknown. In the study, we found that 33O and 42O exhibited distinct developmental competences after IVF, SCNT and PA. Additionally, 18 differentially expressed proteins between 33O and 42O were identified by MALDI-TOF/MS in porcine oocytes.

Insufficient maturation of oocyte is not enough to support the subsequent embryo development, and oocyte will be senescent if overmatured [23]. It has been generally considered that porcine oocyte reaches full maturation at 42 h of IVM, and a number of research groups including us use oocytes matured at 42 h for IVF, PA and SCNT study [17–21]. In the study, we demonstrated the rate of 1^st^ polarbody extrusion of porcine oocytes reached a plateau at 33 h of IVM and the most part of the oocytes arrested at the MII stage. The result was not consistent with a previous report that described porcine oocyte arrived at MII stage during 34.4-48 h of IVM [24], and this may result from the addition of EGF to the IVM medium in the experiments for this research, which may have accelerated the nuclear maturation as already observed in other study [25].

Oocyte maturation is among the most crucial factors affecting embryo development [26–29]. So, we described the effects of 33O and 42O on embryo development of IVF, SCNT and PA. In consistent with previous reports [26–28], the developmental competence of SCNT and PA embryos by 42O were markedly improved in comparison with 33O, indicating that full cytoplasmic maturation is indispensable for development of SCNT and PA embryos. However, surprisingly, 33O was able to sustain IVF embryo development with higher competence comparing to 42O. This is in line with previous results showing that a suitable culture period for IVM of porcine oocytes to IVF is 32 to 36 h [30]. These results could be explained by the following reasons: firstly, according to our results, the rate of fertilization of 33O was higher, so more zygotes may initiate subsequent embryo development; secondly, the nuclear reprogramming of sperm and somatic cell undergoes two distinct processes. The success of SCNT depends on several parameters that impact on the ability of oocyte to remodel the nuclear morphology of donor cell, to reverse the epigenetic modifications to totipotent status, and to activate the embryonic gene expression required for further development [31]. So, the great requirement of reprogramming factors for SCNT must be different with IVF and 42O is rich in these factors [32, 33]; thirdly, the stress-coping mechanisms are probably relying on different molecules between SCNT and IVF embryos. This notion is supported by the fact that SCNT embryos exhibit higher rates of apoptosis, weaker stress-coping functions and are less tolerant to the *in vitro* culture environment than IVF embryos [34]. Lower level of gene expression related to cell protection from stress and apoptosis has been reported in oocyte with low cytoplasmic maturation quality [35], such as SOD1, one of the major antioxidant enzymes, which was proved to scavenge superoxide during early embryonic development [36, 37], which was found abundantly in 42O by our proteome analysis. Considering that, it is reasonable to deduce that transferred somatic nuclei undergo lower cellular stress than sperms in 42O, so that 42O can support better development of SCNT embryos.

During the last decade, several approaches analyzing the protein composition of oocyte have been conducted including the proteome signature of porcine oocyte [6–15], however, the disadvantage of these proteome analyses is that no factor related to nuclear reprogramming and embryo development has been described. In the study, efforts have been made to discover differentially expressed proteins between 33O and 42O by proteome analysis. 994 unique protein spots were detected by 2D DIGE, and only 25 protein spots were found to be differentially expressed between 33O and 42O. That is to say, the difference was no more than 2 % (25/994), indicating the proteome signature of 33O and 42O were very similar. But the developmental competence of 33O and 42O were largely different, suggesting that the few differential proteins identified played important roles in embryo development. This is in line with previous results that only a few proteins which are indispensable for embryo development are largely synthesized from stored mRNAs in the final stage of oocyte maturation [32, 33].

To further clear the molecular basis for cytoplasmic maturation, we focused on function analysis of these identified proteins. Proteins, such as HSP90B1, HSPA5 and HSP90AB1, with chaperone function were most commonly found, reflecting a marked change of nuclear proteins during oocyte maturation [7, 13]. Cluster analysis of the 18 proteins with the UniProt database showed a clear trend towards molecular functions and biological processes related to protein synthesis, including ribonucleotide binding, nucleotide binding and cellular homeostasis, that comfirm the points that accumulation of proteins happen constantly during cytoplasmic maturation [7, 13]. Moreover, the influence of the differential expression factors on development of IVF and SCNT embryos was unraveled by pathway analysis of these proteins using DAVID and Agilent Literature Search. INPP1 and PDIA3 are predominantly involved in fertilization and were found to be over-expressed in 33O. Also, a characterized nuclear reprogramming factor, PARK7, was more abundant in 42O [22]. This led us to suggest that the 18 identified proteins were mainly responsible for nuclear reprogramming and early embryo development.

## Conclusions

In summary, the study identifyed the differentially expressed proteins between porcine oocytes during 33 h and 42 h of IVM. We showed that majority of porcine oocytes have achieved nuclear maturation at 33 h of IVM, and there are 18 protein factors related to nuclear reprogramming and embryo development discovered by proteome analysis of 33O and 44O. Analysis of these factors indicates several of them may be responsible for the development competence difference between 33O and 42O when used for IVF and SCNT. Our study provides valuable information to investigate the functions of maternal proteins. Such efforts will enable us to better understand the molecular mechanism of nuclear reprogramming and embryo development.

## Additional file

Additional file 1: Figure S1. Representative pictures of 33O and 42O with and without zona pellucida. **Table S1.** Details of primers used for Realtime PCR analysis. **Table S2**. The rates of porcine oocyte polarbody extrusion at 33h and 42h of IVM. **Table S3**. The effect of 33O and 42O on IVF. **Table S4**. The enucleation rates of 33O and 42O. **Table S5**. The pronuclear rates of 33O and 42O at 6h after artificial activation.

## Abbreviations

SCNT: Somatic cell nuclear transfer
PA: Parthenogenetic activation
IVF: *In vitro* fertilization
IVM: *In vi*tro maturation
42O: Porcine oocytes with 1st polarbody during 42 h of IVM
33O: Porcine oocytes with 1st polarbody during 33 h of IVM
GV: Germinal vesicle
GVBD: Germinal vesicle breakdown
MII: Metaphase II
iPS: Induced pluripotent stem cells
HSPs: Heat shock proteins
MS: Mass spectrometry
2D DIGE: Two-dimensional difference gel electrophoresis
