# Supplementary material for: Discovery of porcine maternal factors related to nuclear reprogramming and early embryo development by proteomic analysis
Source: Proteome Sci. 2015 Jun 27;13:18. doi: 10.1186/s12953-015-0074-5 (PMC4493956; doi:10.1186/s12953-015-0074-5)
Supplement: Additional file 1: Figure S1. — Representative pictures of 33O and 42O with and without zona pellucida. Table S1. Details of primers used for Realtime PCR analysis. Table S2. The rates of porcine oocyte polarbody extrusion at 33h and 42h of IVM. Table S3. The effect of 33O and 42O on IVF. Table S4. The enucleation rates of 33O and 42O. Table S5. The pronuclear rates of 33O and 42O at 6h after artificial activation. [file 12953_2015_74_MOESM1_ESM.docx]

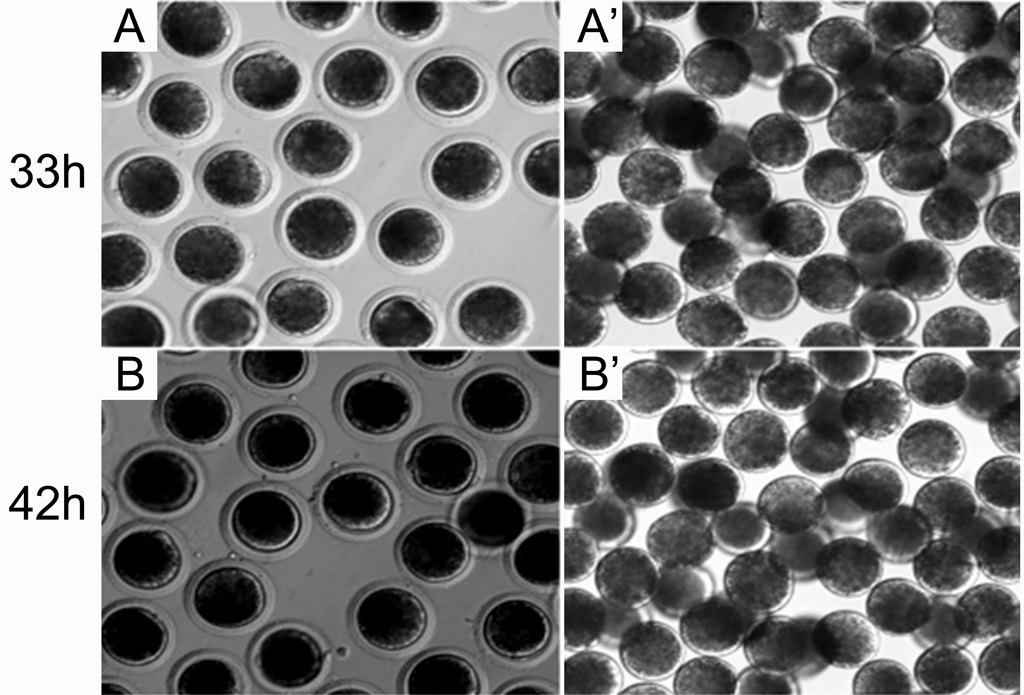


Figure S1: Representative pictures of 33O and 42O with and without zona pellucida.

Highlights:

1. The majority of porcine oocytes achieve nuclear maturation at 33h of IVM and proceed with cytoplasmic maturation from 33h to 42h.
2. Procine oocyte during 33h of IVM is able to sustain IVF embryo development, and further cytoplasmic maturation is indispensable for development of SCNT and PA embryos.
3. 18 differentially expressed proteins between oocytes during 33h and 42h of IVM are identified by mass spectrometry.

**Supplementary TABLE S1. Details of primers used for Realtime PCR analysis.**

| Gene | Accession number | Primer sequences | Product size (bp) |
| --- | --- | --- | --- |
| SOD1 |  | F:TTGGAGACCTGGGCAATGTGAC  R: CTTCCAGCATTTCCCGTCTTTGTA | 184 |
| MVP |  | F: ACGGCAAGGAGAGGGTGACAG  R: CGCCTACCACCTCCTCATAGACAT | 252 |
| INPP1 |  | F: CTCAGGTGGAAAGGACAGTGCTAC  R: TGAGAACGGGTGCGGGC | 156 |
| PARK7 |  | F: GGAGCAGAGGAGATGGAGACGG  R: GCTGGCGTCGGGACAAATG | 135 |
| EEF1A1 |  | F: CCTGGGTCTTGGACAAACTAAAGG  R: CGTTCTTGGAAATACCTGCTTCG | 222 |
| Gstm2 |  | F: CTTTGAGAAACTGAAGCCTGGGTAC  R: GGATCGAATATGCGGTACAGGTC | 159 |
| HSPA5 |  | F: CACTTGGTATTGAAACCGTGGG  R: CATAGACCTTGATGGTAACAGTTGG | 132 |
| VIM |  | F: CTTCAGGAGGCGGAGGAGTGG  R: CTGCACGCGGCCAATAGTGTC | 249 |
| NUDT5 |  | F: AAGGACAGTCGGCTGATGGC  R: CCTTGTAGCCAGTCTCTTCCTCG | 198 |
| PDIA3 |  | F: TGCTGGAACTCACCGACGAC  R: TACAATCAACCTTTGCTAATGGAACTA | 177 |
| HSPAA1 |  | F: GGAAATCGCCCAGTTGATGTCG  R: CCACTATCGTGAGGGTCCGGTC | 206 |
| PRSS1 |  | F: CTGAAGGCTCCCGTCCTAAGTG  R: CCTGGCAAGAATCCTTTCCACC | 112 |
| PADI6 |  | F: GATGGCAAATGACAAACAGGCTAAG  R: TGGAGGTGTGGAGCAGCAGC | 227 |
| 18s rRNA |  | F: TCCAATGGATCCTCGCGGAA  R: GGCTACCACATCCAAGGAAG | 149 |

**Supplementary TABLE S2. The rates of porcine oocyte polarbody extrusion at 33h and 42h of IVM**

| Culture period | No. oocytes (repeats) | The rate of polar body extrusion  (% ± SEM) |
| --- | --- | --- |
|  |  |  |
| 33h | 100 (3) | 77 (76.72±2.29) |
|  |  |  |
| 42h | 101 (3) | 83 (81.80±1.86) |

**Supplementary TABLE S3. The effect of 33O and 42O on IVF**

| culture period | No. oocytes | Fertilization (%±SEM) |
| --- | --- | --- |
|  |  |  |
| 33h | 77(3) | 65 (85.19±1.49)^a^ |
| 42h | 62(3) | 49 (78.09±2.72)^b^ |

Note: Values with different superscripts within columns differ significantly (*P*<0.05).

**Supplementary TABLE S4. The enucleation rates of 33O and 42O**

| Culture period | Repeats | No. oocytes | No. oocytes enucleated (% ± SEM) |
| --- | --- | --- | --- |
| 33h | 3 | 93 | 90(99.12±0.88)^a^ |
| 42h | 3 | 79 | 73(91.87±2.46)^b^ |

Note: Values with different superscripts within columns differ significantly (*P*<0.05).

**Supplementary TABLE S5. The pronuclear rates of 33O and 42O at 6h after artificial activation**

| Culture period | No. oocytes (repeats) | No. pronucleus  (% ± SEM) |
| --- | --- | --- |
| 33h | 107 (3) | 89 (83.78±7.04) |
| 42h | 110 (3) | 100 (91.43±6.78) |
